# Measurement of skin dose from cone-beam computed tomography imaging

**DOI:** 10.1186/1746-160X-9-28

**Published:** 2013-10-09

**Authors:** Sercan Akyalcin, Jeryl D English, Kenneth M Abramovitch, Xiujiang J Rong

**Affiliations:** 1 Department of Orthodontics, The University of Texas Health Science Center at Houston, School of Dentistry, Houston, TX, USA; 2 Oral Diagnosis, Radiology, and Pathology, Loma Linda University, School of Dentistry, Loma Linda, CA, USA; 3 Department of Imaging Physics, MD Anderson Cancer Center, Houston, TX, USA

**Keywords:** Skin dose, Cone-beam computed tomography, OSL dosimeters

## Abstract

**Objective:**

To measure surface skin dose from various cone-beam computed tomography (CBCT) scanners using point-dosimeters.

**Materials & methods:**

A head anthropomorphic phantom was used with nanoDOT optically stimulated luminescence (OSL) dosimeters (Landauer Corp., Glenwood, IL) attached to various anatomic landmarks. The phantom was scanned using multiple exposure protocols for craniofacial evaluations in three different CBCT units and a conventional x-ray imaging system. The dosimeters were calibrated for each of the scan protocols on the different imaging systems. Peak skin dose and surface doses at the eye lens, thyroid, submandibular and parotid gland levels were measured.

**Results:**

The measured skin doses ranged from 0.09 to 4.62 mGy depending on dosimeter positions and imaging systems. The average surface doses to the lens locations were ~4.0 mGy, well below the threshold for cataractogenesis (500 mGy). The results changed accordingly with x-ray tube output (mAs and kV) and also were sensitive to scan field of view (SFOV). As compared to the conventional panoramic and cephalometric imaging system, doses from all three CBCT systems were at least an order of magnitude higher.

**Conclusions:**

Peak skin dose and surface doses at the eye lens, thyroid, and salivary gland levels measured from the CBCT imaging systems were lower than the thresholds to induce deterministic effects. However, our findings do not justify the routine use of CBCT imaging in orthodontics considering the lifetime-attributable risk to the individual.

## Introduction

A three-dimensional radiographic examination of the craniofacial skeleton with cone beam computed tomography (CBCT) is indicated for a number of clinical conditions. However, like any x-ray exposure, CBCT scans also expose the patient to certain biologic risks of radiation. As the indications for CBCT imaging become more universal, so does the concern for radiation safety related to dental and orthodontic procedures [1-3].

In diagnostic imaging, exposure to x-ray radiation must be accompanied by a related benefit that outweighs the associated risks for the use of that radiation. Orthodontists, as practicing health-care providers, must remain cognizant of the risks if CBCT imaging is to become a more integral part of standard orthodontic practice. If so, it is important to know what the radiation doses are for orthodontic-indicated CBCT scans. There are already advocates for the universal use of CBCT scans to replace conventional radiographs. Their claim is based on the premise that the radiation doses from CBCT are lower than the combined radiation dose of a lateral cephalogram, panoramic radiograph and a full series of periapical radiographs [2,4]. However, there is no conclusive evidence to fully support these views.

In CT radiation dosimetry, CT Dose Index (CTDI) and its variations such as CTDI_100_, CTDI_W_, and CTDI_vol_[5-7] have been used widely in comparing dose levels of different scanners and for the purpose of quality assurance. As its name states, CTDI is a dose descriptor, not a direct measurement of patient dose. Because it is measured by using a standardized, homogeneous, cylindrical phantom, it questionably represents the dose for objects of substantially different size, shape, or attenuation, like the human body [7]. Additionally, in the case of cone-beam geometry; the CTDI concept is no longer valid because of its wide-open beam. An alternative method has to be determined for representing radiation dose in CBCT scans. Ideally, in order to determine the dose to a point within the scan volume, a point (small) dosimeter is required. For such evaluations optically stimulated luminescence (OSL) 'dot’ dosimeters, thermoluminescent dosimeters (TLDs), small solid-state detectors, and metal oxide semiconductor field-effect transistors (MOSFET) have been used. It was recently concluded that OSL dot dosimeters had good reproducibility and stability in both laboratory and field-tests and met the performance requirements of standards of the American National Standards Institute [8].

Radiation exposure puts the patient at risk of getting a radiation-induced cancer or heritable mutation, i.e., stochastic effect. To assess the patient radiation risk from a radiation-protection perspective, the effective dose unit of measurement is regarded as the most suitable dose index [9]. Effective dose takes into account the types of tissues being exposed and the amount of radiation dose to each tissue. It attempts to reflect the equivalent whole-body dose that results in a stochastic effect, which is equivalent to stochastic effect from the actual absorbed dose to those tissues irradiated in a non-uniform, partial body irradiation such as a CT scan [7].

Effective dose (Sv) is calculated by a formula that uses measured absorbed tissue/organ doses exposed during a radiographic procedure and the tissue weighting factors determined based on the radiosensitivity of each organ (ICRP 103). Research studies [10-17] using human phantoms have overly reported the dose estimation from dental CBCT in Sv which is a unit of measure of effective dose for the estimation of whole-body risk in the context of stochastic detriment at low doses instead of Gy which is a hard-physical concept to be used for local radiation absorbed doses at these localized sites. Moreover, the effective dose from dental CBCT is typically low when compared to other medical CT scans mainly because dental CBCT is limited to exposing only the head; and the weighting factors of the organs in the head are relatively small. Calculations of exposure to more radiosensitive areas and larger areas such as gonadal tissues, breast, colon, lung and stomach are not considered. Although the radiation exposure from a dental CBCT is isolated to a portion of head, this area is usually repeatedly exposed. There is little or no published data on measured skin doses with the use of dental CBCT.

The objective of this study was to directly measure skin dose using OSL nanoDOT dosimeters from multiple operational scanning modes of three CBCT scanners and to compare them to similarly measured skin doses from conventional panoramic and cephalometric imaging.

## Materials and methods

Four dental x-ray imaging systems were investigated in this study: three CBCT units and one conventional combined panoramic-cephalometric x-ray unit. The CBCT units were the Kodak 9500 (Kodak Dental Systems, Carestream Health, Rochester, NY, USA), i-CAT Next Generation (Imaging Sciences International, Hatfield, PA, USA), Galileos Comfort (Sirona Dental Systems, Bensheim, Germany). The conventional unit was the ProMax pan/ceph x-ray unit (Planmeca U.S.A. Inc., Roselle, IL). CBCT scanners were operated under multiple scanning modes. Scan settings are listed in Table 1. Scan protocols were grouped by scan field of view (SFOV) size. Only medium and large SFOVs were included in the study since they are the most commonly used ones in orthodontic diagnosis and treatment planning. Volume diameters or cylinder heights between 10-16 cm were classified as 'medium’ and those greater than 16 cm were placed in the 'large’ category (Table 1).

**Table 1:** Scan settings of the CBCT and conventional x-ray imaging units used in the study

**X-ray unit**	**Scan protocol**	**Tube voltage (kV)**	**mAs**	**Scan time (s)**	**sFOV (cmxcm)**	**Definition (voxel)**
Kodak 9500	Medium	86	108	10.8 sec	15X9	0.2
Kodak 9500	Large	120	108	10.8 sec	20X18	0.3
iCAT Next Generation	Medium	120	18.54	8.9 sec	16X13	0.4
iCAT Next Generation	Medium	120	20.27	14.7 sec	16X13	0.25
iCAT Next Generation	Large	120	18.54	8.9 sec	23X17	0.3
iCAT Next Generation	Large	120	37.07	17.8 sec	23X17	0.3
Galileos Comfort	Medium	85	21	14 sec	15x15	0.3
Galileos Comfort	Medium	85	42	14 sec	15x15	0.3
ProMax panoramic	Standard panoramic 25X30	66	144	16 sec		
ProMax cephalometric	Full cephalogram 30X27	68	112.2	18.7 sec		

**Figure 1:**
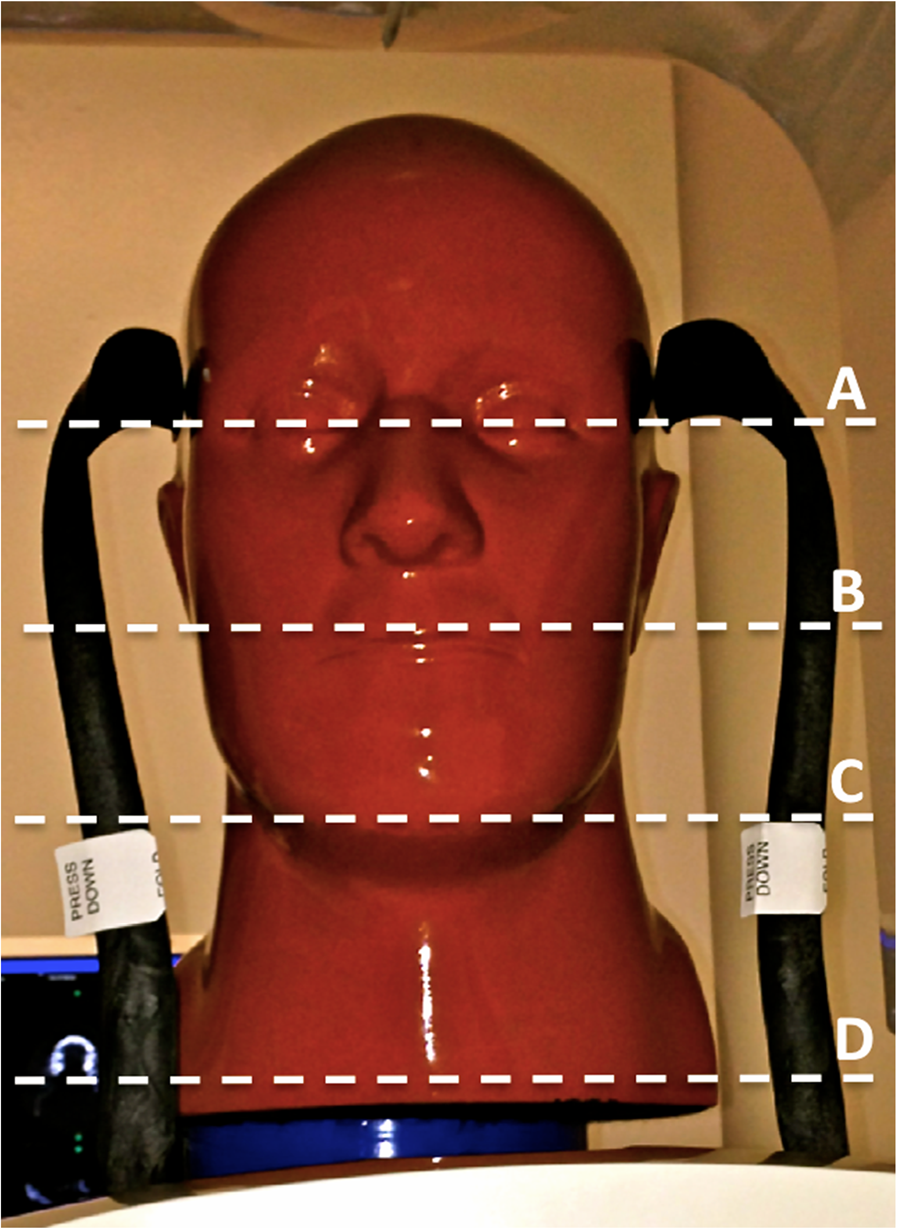
**RS-110 anthropometric head phantom used in this study with the levels where nanoDOTS were located. A**- Eye lens **B**- Parotid gland **C**- Submandibular gland **D**- Thyroid gland.

**Figure 2:**
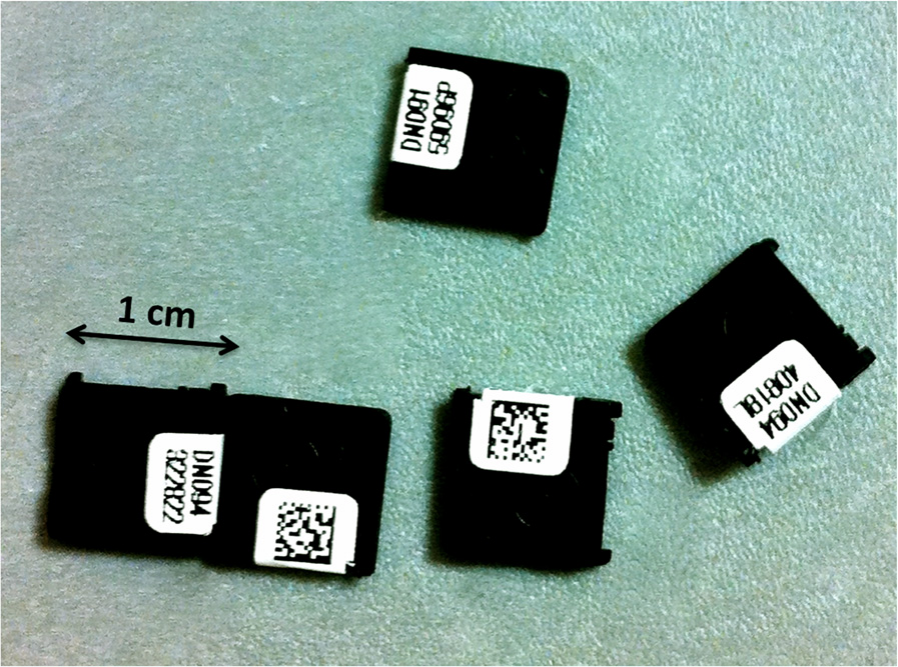
nanoDOT OSL dosimeters (Landauer Corp., Glenwood, IL).

A head anthropomorphic phantom-- RS-110 (Radiology Support Devices (RSD) Inc., Long Beach, CA) (Figure 1) was used with nanoDOT dosimeters (Landauer Corp., Glenwood, IL) (Figure 2) attached to the surface area at the levels of following anatomic landmarks: eye lens, parotid, submandibular, and thyroid glands. RSD phantoms are constructed with skeletons that meet radiation interaction properties of both cortical bone and spongiosa as standardized by the International Commission on Radiation Units and Measurements (ICRU). Moreover, soft-tissue molds and skeleton molds are matched for anatomic fidelity and to simulate attenuation characteristics of an average adult human male subject. Specified radiosensitive tissues of interest were chosen to provide multiple skin dose readings of the craniofacial area and to also compare those to the threshold limits of the related organ doses at the investigated regions. The peak skin dose was computed by selecting the highest value of absorbed dose among the investigated radiosensitive regions of interest; eye lens, parotid, submandibular, thyroid doses were computed by averaging the absorbed dose for nanoDOTS tested within that region.

**Figure 3:**
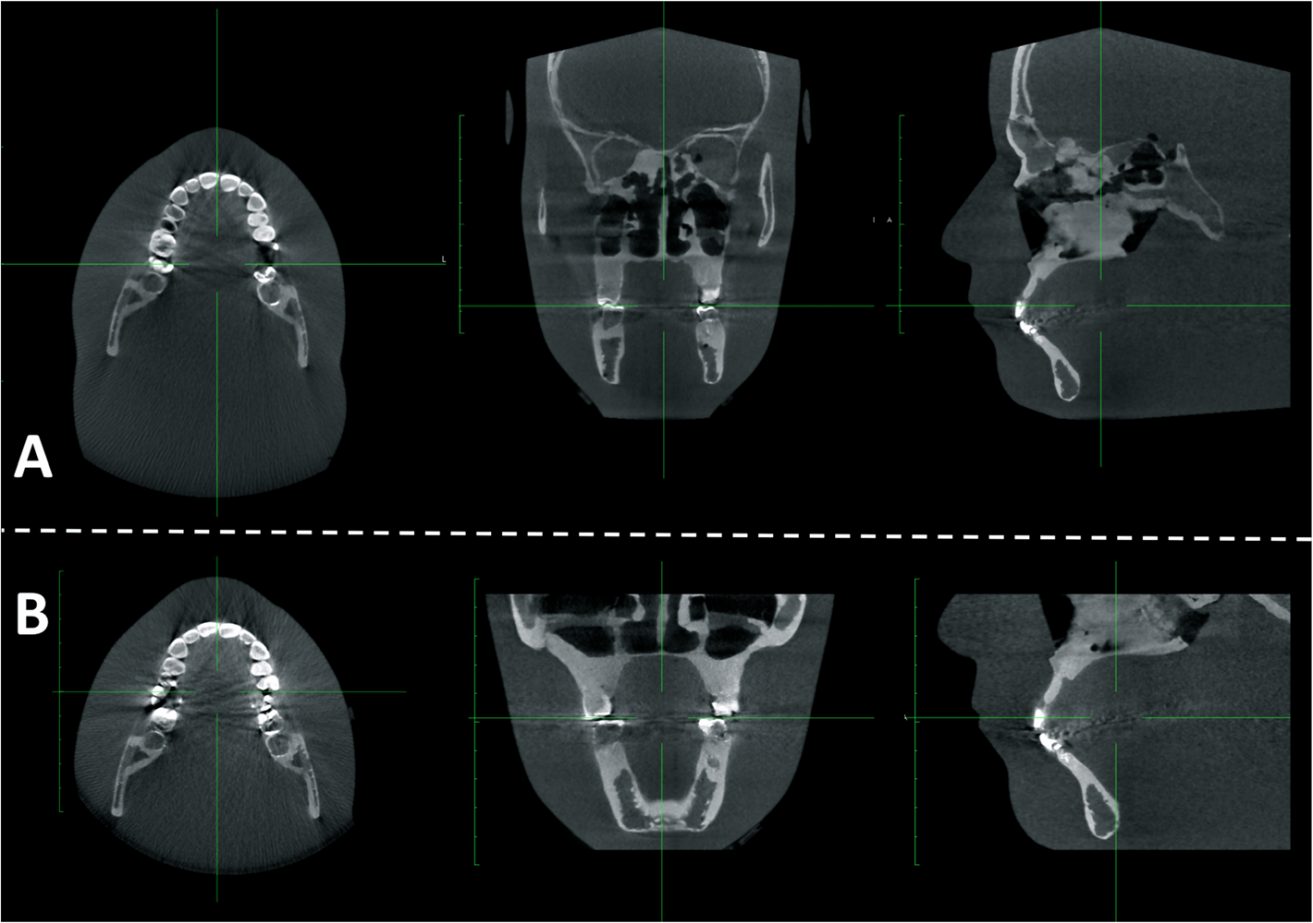
Example of the axial, coronal and sagittal images obtained with KODAK 9500 scanner using the head anthropomorphic phantom: a- Medium FOV b- Large FOV.

The phantom was positioned with the midsagittal plane in the center of each image and the occlusal plane parallel to the scan rotation plane (Figure 3). Three individual nanoDOTS were used for each of the image scans at each scan site. This was done to compare the reliability of the dose readings using intraclass coefficients (ICCs). Exposed nanoDOTS were processed using a microStar reader (Landauer Corp., Glenwood, IL). The counts read out from the microStar reader were converted to absorbed dose in milligrays (mGy) using unit and scan protocol specific calibration factors. The calibrations were performed for each unit and the energies (kVp) selected for dose measurements. During a calibration, 2-3 nanoDOTs were placed next to a small volume ion chamber (10X5-0.6 Modern Wide Beam Multi-Slice CT Chamber, RadCal Corporation Monrovia, CA) in air and at the isocenter of the scan rotation. A RadCal MDH model 1515 electrometer was used to read out signals from the ion chamber. This onsite user calibration minimizes energy dependence of the nanoDOTs and hence ensures accurate dose measurements throughout this study. It is critical to perform such calibrations for each system since the vendor calibration was performed using a general radiographic unit with energy spectra and beam conditions significantly different from CBCT units.

## Results

ICCs derived from three sets of nanoDOTS ranged between 0.97-0.99 and indicated a high level of accuracy. Therefore, the average of the three exposures was used for the individual dose measurements. The peak skin dose and doses at the investigated landmarks were calculated as absorbed dose and were presented in Table 2. The skin doses ranged from 0.09 to 4.62 mGy. The wide range was caused by the differences between the CBCT scanners and the conventional x-ray unit as well as the differences in imaging protocols such as kilovolt (kV) and the tube current-time product (mAs), especially scanning field of view (sFOV). iCAT scanner had consistently lower doses for all the variables as compared to other scanners.

**Table 2:** Peak skin and absorbed tissue doses for the investigated scan protocols

**X-ray unit**	**kV**	**mAs**	**sFOV**	**Skin**	**Eye lens**	**Parotid**	**Submandibular**	**Thyroid**
				**Dose (mGy)**	**Dose (mGy)**	**Dose (mGy)**	**Dose (mGy)**	**Dose (mGy)**
Kodak 9500	86	108	15×9	3.58	0.42	2.88	3.04	0.32
Kodak 9500	120	108	20×18	4.15	3.55	3.57	3.17	0.45
iCAT Next Generation	120	18.54	16×13	1.44	0.92	1.15	1.21	0.24
iCAT Next Generation	120	20.27	16×13	2.51	1.60	2.05	1.79	0.25
iCAT Next Generation	120	18.54	23×17	2.08	1.41	1.93	1.79	0.25
iCAT Next Generation	120	37.07	23×17	2.60	1.75	2.11	2.23	0.39
Galileos Comfort	85	21	15×15	2.49	0.58	2.45	1.93	0.36
Galileos Comfort	85	42	15×15	4.62	0.94	4.46	3.61	0.46
ProMax panoramic	66	144		0.26	0.05	0.24	0.14	0.04
ProMax cephalometric	68	112.2		0.09	0.07	0.06	0.06	0.05

The surface doses to the locations of the eyes were ~4.0 mGy, well below the 500 mGy threshold for possibly causing cataract development. Relatively higher radiation doses were recorded for submandibular and parotid regions in all the CBCT protocols. Lowest doses were obtained for the thyroid region partly due to the fact that this area is not covered by the sFOV and therefore is not directly exposed. Similar results were obtained for the eyes in medium size sFOV protocols. Conventional radiographs had the lowest doses for all of the variables tested. However, slight differences between the cephalometric and panoramic images were observed due to their coverage field as expected.

## Discussion

Dental cone beam computed tomography (CBCT) has rapidly gained popularity among the dental specialties over the last decade. It is a reality that individuals that require the investigation of the maxillofacial structures in all three dimensions of the space, as usually is the case in orthodontics and maxillofacial surgery, will benefit from CBCT imaging. Although there is a lack of definitive data, recent reviews signify the importance of radiation dose generated by CBCT scanning as a cause for concern [1-4,18,19]. News media is very sensitive on the unjustified use of CBCT scans on young children and adolescents in the orthodontic practice [20]. A recent article [21] that reported links between some dental x-rays and an increased risk of intracranial meningioma has become a public sensation and has led professionals to question the applicability of the presented data to other dental diagnostic tools, which depend on more radiation dose including CBCT.

In reality, it is extremely difficult to determine the risks of using CBCT scanners in terms of fatal cancer development because of the confounding factors in dose estimation such as individual differences in patients’ physical attributes, biological susceptibility and challenges with dose estimation. However, without dose measurements operators lack the objective data needed to approximately adjust mAs or tube potential in order to avoid excessive patient dose [7].

This study mainly focused on the peak skin dose together with the surface entry doses for various other radiosensitive tissues/organs. The rationale was to compare various operational modes of multiple CBCT scanners with each other and more importantly with the conventional radiographs that are routinely used in orthodontic treatment. Based on the investigated CBCT protocols, peak skin dose at any point did not exceed 4.62 mGy, which is well below 1000 mGy. The International Commission on Radiological Protection (ICRP) guidelines suggest that 2000 mGy may cause transient erythema and temporary epilation [22-24]. In medical imaging, 2000 mGy is also regarded as the threshold for deterministic effects [25,26]. However, for most patients, clinically important skin and hair reactions occur only when the skin dose is higher than 5000 mGy [27]. As evidenced in this paper, CBCT exposure for orthodontic exams is less than 2.5% that of skin threshold dose.

Although there is a wide variation in our results for the skin dose due to different scanning protocols, one cannot claim the superiority of one scanner over another solely based on the reported dose information. Each CBCT scanner has different settings and energy levels. Perhaps, the use of pulsed x-ray beam exposure in this study was also a major reason for considerable variation in reported cone-beam unit dosimetry. However, our results in Table 2 clearly demonstrate that when all the other factors were held constant, mAs, kV, and sFOV settings had effects on the observed radiation doses, with mAs setting being the most effective. Despite the fact that iCAT scanner had a higher kV setting, consistently lower doses were observed in all the iCAT scanner protocols compared to the others. This may be explained by lower mAs settings of this device. Similarly, when the mAs setting of the Galileos was increased from 21 to 42, peak skin dose increased almost 2 times. As higher tube currents are often used for larger patients to maintain image quality, this finding suggests that mAs value should be kept minimum wherever possible as long as there is no significant compromise in the image quality. In computed radiography overexposure will only reduce image noise and can occur without awareness, as CBCT images never look overexposed. This is due to the normalization process of patient attenuation with the CBCT technique. Therefore, operators need to be informed about the purpose of the scan before they set the tube current to the default mode or to a higher setting unless otherwise is instructed.

Radiation dose at the level of the eyes ranged between 0.42 to 3.55 mGy. Current permissible exposure limits to eyes are similar to the skin dose [23,24]. However, according to newer studies the threshold for cataractogenesis is actually much lower. Recently published data on Chernobyl cleanup workers revealed a significant increase in cataract rates with increasing radiation doses, which were, for the most part, less than 500 mGy [28]. Additionally, Chodick *et al*. [29], argued that likelihood of cataract formation increased with increasing exposure to ionizing radiation with no apparent threshold level. On the contrary, it was also suggested that there was no association between computed tomography scans of the head history and cataract [30]. The evidence from the literature seems to be inconclusive on the radiation induced cataract development. However, there is still 140 times less risk with the maximum dose obtained with the diagnostic CBCT protocols used in this study when compared to the recent Chernobyl data [28]. Additionally, sFOV may be changed from large to medium, depending on the imaging need, to avoid the exposure of the eyes when monitoring the jaws only. There will still be scattered radiation as is the case with thyroid gland. Our results demonstrated that radiation dose at the thyroid level had the least variability since it is not within direct exposure field. Even then a dose range of 0.24-0.46 mGy was observed at that level that can be considered as low. Additionally, Qu *et al *[31]. recently demonstrated that with the use of thyroid collars, dose to thyroid and oesophagus could effectively be reduced to 48.7% and 41.7%, respectively. It was also shown that the radiation dose to the eye could be reduced by over 60% through the use of leaded glasses during a CBCT examination [32].

One of the unique aspects of the current study was to utilize the Optically Stimulated Luminescence (OSL) 'dot’ dosimeters. It was already shown that single-irradiation measurements with bleaching after each OSL readout was found to be associated with a 3.3% reproducibility [33]. In our study, we were also able to observe high reproducibility for dose measurements with the use of OSL dots. In order to compare conventional radiographs to CBCT scanners, absorbed dose was preferred to a whole-body effective dose, which would require too many assumptions since dental CBCT only exposes the head region. Reported values enable a sound comparison between the procedures for the investigated parameters. Relatively much lower point doses were obtained with both the panoramic and cephalometric modes of ProMax unit. It should, however, be remembered that dose differences are due to the differences in geometry and specific parameters of the CBCT scanners. While the dose range is too wide between the conventional radiographs and CBCT scanners, too much attention to radiogenic risk may also distract attention from other risks and potential benefits, which may not be in the patient’s best interest [34]. In that sense it is not proper to misrepresent an individual’s radiation history as part of the risk of the proposed procedure. Due to the very recent advisory statement by the American Dental Association Council on Scientific Affairs [35], only a trained clinician must decide if a procedure can be justified by itself on the basis of radiation and other risks of that procedure, the patient’s clinical status, and the benefits expected from that procedure. Although doses reported in this paper may be perceived as very low when compared to those in medical imaging, a lifetime-attributable risk to the individual should also be considered.

## Conclusions

When planning orthodontic treatment, conventional panoramic and cephalometric radiographs are certainly dose sparing when compared to CBCT scans. However, when indicated, CBCT imaging should be considered with a radiation conscious approach. As evidenced in this paper, scan parameters such as mAs and FOV settings of the CBCT scanners should be used effectively depending on the imaging and individual patient needs for dose reduction purposes. Hence features of variable kVp stations, mAs selections, and radiation beam collimation settings are preferable to a well designed CBCT system for dental imaging.

## Abbreviations

CBCT: Cone-beam computed tomography; OSL: Optically stimulated luminescence; SFOV: Scanning field of view; CTDI: CT Dose Index; TLDs: Thermoluminescent dosimeters; MOSFET: Metal oxide semiconductor field-effect transistors; ICRU: International Commission on Radiation Units and Measurements; ICRP: The International Commission on Radiological Protection.

## Competing interests

The authors declare that they have no competing interests.

## Authors’ contributions

SA and XJR were responsible for the design and they carried out the experimental part of the project. They both oversaw all phases of the project. JDE performed the dose measurements and helped with the data interpretation. KMA participated in the design, drafted the paper and provided feedback on the writing. All authors read and approved the final manuscript.
